# Thirty-Eight Cases

**Published:** 1845-01

**Authors:** Robert P. Harris

**Affiliations:** Resident Physician


WILL'S HOSPITAL.

Service of DR. HAYS.

| | | | |
|---|---|---|---|
| Patients in the House, November 1st, 1844, | | | 22 |
| " admitted during November, Males | 5 | | |
| " " " " Females | 3 | | |
| | | — 8 | |
| " discharged " " Males | 4 | | |
| " " " " Females | 3 | | |
| | | — 7 | |
| " in the House at the end of the month, | | | |
| " " " " " " Males | 18 | | |
| " " " " " " Females | 5 | | |
| | | —23 | |
| " with diseases of the eyes | 21 | | |
| " " " " limbs | 2 | | |
| Total in the House, November 30th, | | —23 | |

Out-door Patients, Males 4, Females 4.

*Operations.*—For Cataract, 1.

ROBERT P. HARRIS, M. D.,
Resident Physician.

*Ibid.*

